# Supplementary material for: Time series analysis of the demand for COVID-19 related chest imaging during the first wave of the SARS-CoV-2 pandemic: An explorative study
Source: PLoS One. 2021 Mar 3;16(3):e0247686. doi: 10.1371/journal.pone.0247686 (PMC7928469; doi:10.1371/journal.pone.0247686)
Supplement: S1 Table — Number of requests for coronavirus disease 2019 (COVID-19) related chest imagings per day (total, outpatients, in-patients) and number of regional COVID-19 cases per day provided by the Robert Koch Institute [15]. (DOCX) [file pone.0247686.s001.docx]

**S1 Table. Demand for COVID-19 related chest imaging and regional COVID-19 case numbers.**

| Date | Requests total | Requests outpatients | Requests in-patients | Regional cases |
| --- | --- | --- | --- | --- |
| 27.02.2020 | 0 | 0 | 0 | 0 |
| 28.02.2020 | 1 | 1 | 0 | 0 |
| 29.02.2020 | 0 | 0 | 0 | 1 |
| 01.03.2020 | 0 | 0 | 0 | 1 |
| 02.03.2020 | 0 | 0 | 0 | 0 |
| 03.03.2020 | 0 | 0 | 0 | 3 |
| 04.03.2020 | 0 | 0 | 0 | 1 |
| 05.03.2020 | 0 | 0 | 0 | 5 |
| 06.03.2020 | 0 | 0 | 0 | 3 |
| 07.03.2020 | 0 | 0 | 0 | 4 |
| 08.03.2020 | 0 | 0 | 0 | 5 |
| 09.03.2020 | 2 | 0 | 2 | 21 |
| 10.03.2020 | 0 | 0 | 0 | 30 |
| 11.03.2020 | 2 | 2 | 0 | 30 |
| 12.03.2020 | 2 | 1 | 1 | 41 |
| 13.03.2020 | 2 | 2 | 0 | 38 |
| 14.03.2020 | 1 | 1 | 0 | 78 |
| 15.03.2020 | 2 | 2 | 0 | 78 |
| 16.03.2020 | 4 | 3 | 1 | 80 |
| 17.03.2020 | 8 | 5 | 3 | 133 |
| 18.03.2020 | 3 | 2 | 1 | 135 |
| 19.03.2020 | 8 | 7 | 1 | 186 |
| 20.03.2020 | 11 | 6 | 5 | 146 |
| 21.03.2020 | 6 | 3 | 3 | 106 |
| 22.03.2020 | 4 | 2 | 2 | 149 |
| 23.03.2020 | 9 | 4 | 5 | 133 |
| 24.03.2020 | 9 | 7 | 2 | 207 |
| 25.03.2020 | 9 | 5 | 4 | 162 |
| 26.03.2020 | 13 | 8 | 5 | 170 |
| 27.03.2020 | 10 | 4 | 6 | 142 |
| 28.03.2020 | 8 | 6 | 2 | 145 |
| 29.03.2020 | 9 | 5 | 4 | 115 |
| 30.03.2020 | 9 | 7 | 2 | 71 |
| 31.03.2020 | 13 | 4 | 9 | 159 |
| 01.04.2020 | 5 | 2 | 3 | 139 |
| 02.04.2020 | 11 | 4 | 7 | 165 |
| 03.04.2020 | 11 | 6 | 5 | 158 |
| 04.04.2020 | 7 | 3 | 4 | 108 |
| 05.04.2020 | 3 | 3 | 0 | 108 |
| 06.04.2020 | 12 | 2 | 10 | 93 |
| 07.04.2020 | 6 | 4 | 2 | 210 |
| 08.04.2020 | 7 | 1 | 6 | 117 |
| 09.04.2020 | 12 | 5 | 7 | 114 |
| 10.04.2020 | 7 | 3 | 4 | 97 |
| 11.04.2020 | 6 | 3 | 3 | 79 |
| 12.04.2020 | 15 | 6 | 9 | 46 |
| 13.04.2020 | 5 | 1 | 4 | 41 |
| 14.04.2020 | 6 | 1 | 5 | 76 |
| 15.04.2020 | 14 | 4 | 10 | 83 |
| 16.04.2020 | 11 | 2 | 9 | 73 |
| 17.04.2020 | 5 | 0 | 5 | 108 |
| 18.04.2020 | 11 | 2 | 9 | 39 |
| 19.04.2020 | 4 | 2 | 2 | 22 |
| 20.04.2020 | 10 | 3 | 7 | 33 |
| 21.04.2020 | 11 | 2 | 9 | 57 |
| 22.04.2020 | 12 | 2 | 10 | 49 |
| 23.04.2020 | 7 | 0 | 7 | 49 |
| 24.04.2020 | 10 | 2 | 8 | 41 |
| 25.04.2020 | 10 | 2 | 8 | 44 |
| 26.04.2020 | 3 | 0 | 3 | 17 |
| 27.04.2020 | 9 | 0 | 9 | 21 |
| 28.04.2020 | 10 | 3 | 7 | 25 |
| 29.04.2020 | 6 | 0 | 6 | 42 |
| 30.04.2020 | 7 | 0 | 7 | 25 |
| 01.05.2020 | 8 | 3 | 5 | 8 |
| 02.05.2020 | 5 | 1 | 4 | 13 |
| 03.05.2020 | 3 | 0 | 3 | 3 |
| 04.05.2020 | 6 | 0 | 6 | 9 |
| 05.05.2020 | 7 | 1 | 6 | 21 |
| 06.05.2020 | 8 | 2 | 6 | 21 |
| 07.05.2020 | 9 | 4 | 5 | 17 |
| 08.05.2020 | 9 | 4 | 5 | 12 |
| 09.05.2020 | 4 | 0 | 4 | 10 |
| 10.05.2020 | 6 | 3 | 3 | 3 |
| 11.05.2020 | 5 | 0 | 5 | 4 |
| 12.05.2020 | 6 | 1 | 5 | 2 |
| 13.05.2020 | 7 | 0 | 7 | 14 |
| 14.05.2020 | 5 | 2 | 3 | 10 |
| 15.05.2020 | 11 | 3 | 8 | 12 |
| 16.05.2020 | 1 | 0 | 1 | 6 |
| 17.05.2020 | 2 | 0 | 2 | 1 |
| 18.05.2020 | 9 | 0 | 9 | 1 |
| 19.05.2020 | 8 | 0 | 8 | 3 |
| 20.05.2020 | 3 | 0 | 3 | 13 |
| 21.05.2020 | 2 | 0 | 2 | 2 |
| 22.05.2020 | 8 | 0 | 8 | 3 |
| 23.05.2020 | 2 | 1 | 1 | 3 |
| 24.05.2020 | 2 | 1 | 1 | 0 |
| 25.05.2020 | 11 | 0 | 11 | 1 |
| 26.05.2020 | 5 | 0 | 5 | 3 |
| 27.05.2020 | 1 | 0 | 1 | 1 |

Number of requests for coronavirus disease 2019 (COVID-19) related chest imagings per day (total, outpatients, in-patients) and number of regional COVID-19 cases per day provided by the Robert Koch Institute [15].
