# Supplementary figures and images for: Time series analysis of the demand for COVID-19 related chest imaging during the first wave of the SARS-CoV-2 pandemic: An explorative study
Source: PLoS One. 2021 Mar 3;16(3):e0247686. doi: 10.1371/journal.pone.0247686 (PMC7928469; doi:10.1371/journal.pone.0247686)

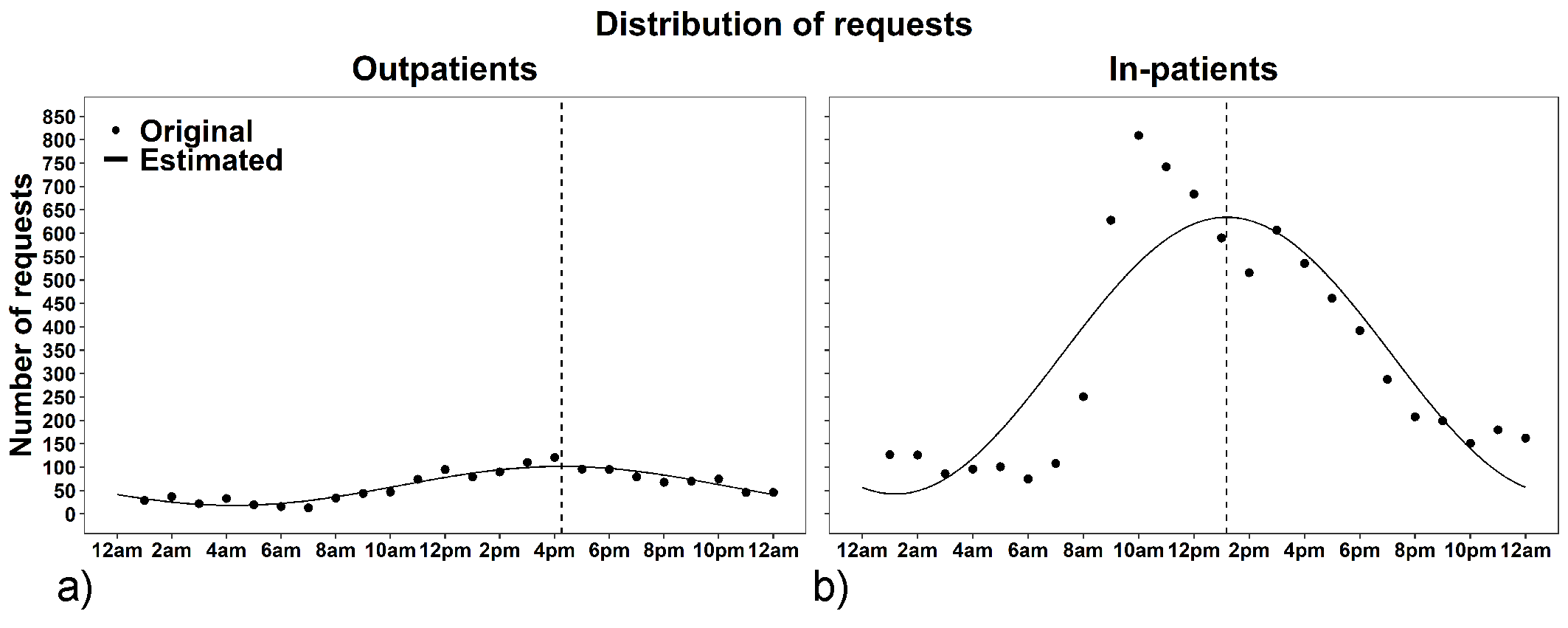

Supplement: S1 Fig — Number of requests for chest imaging of outpatients (a) and in-patients (b) without suspected or confirmed coronavirus disease 2019 (COVID-19) per hour of the day. The dotted lines indicate the acrophases at 4:16 pm (a) and 1:11 pm (b). (TIF) [file pone.0247686.s002.tif]

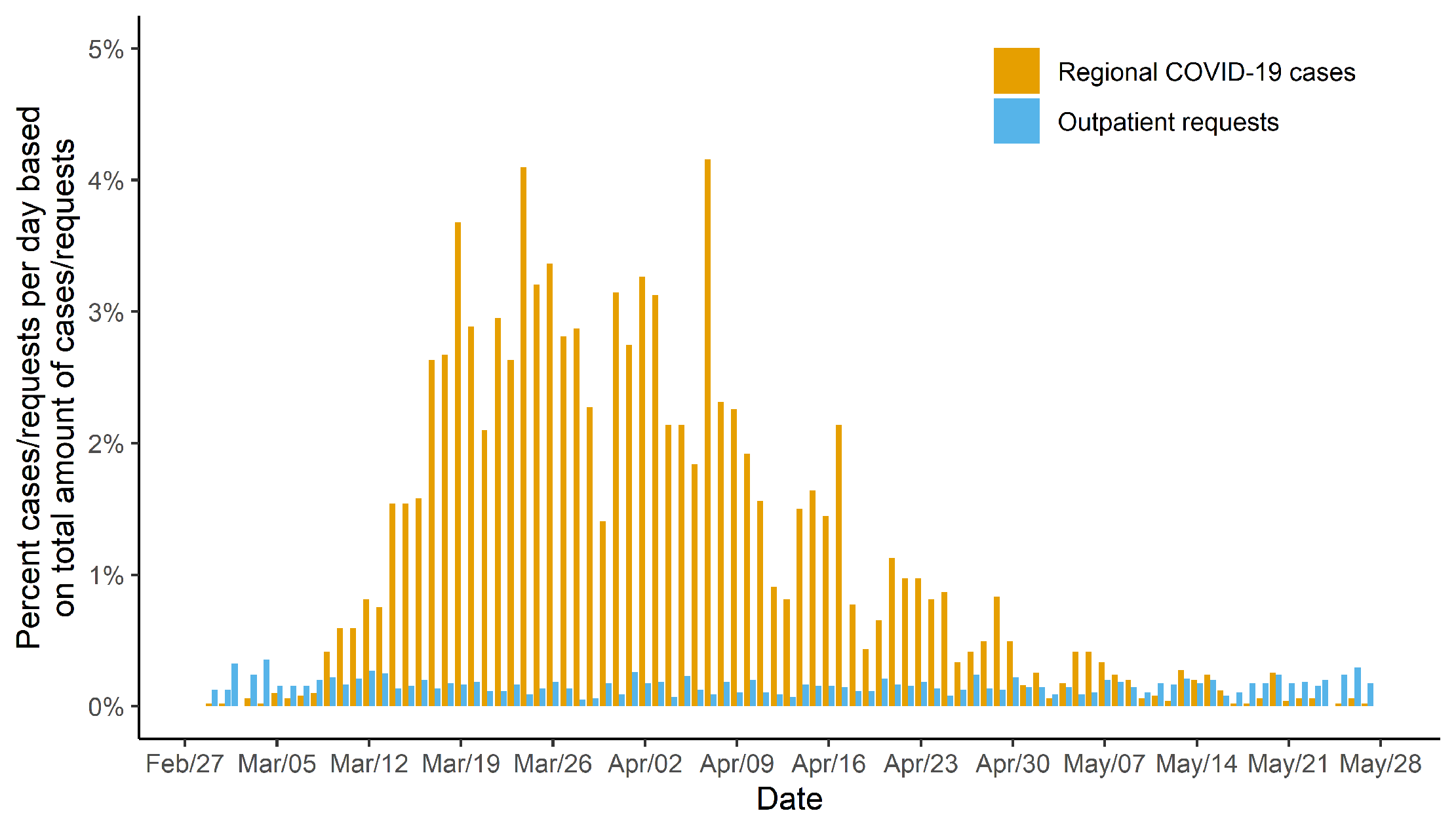

Supplement: S2 Fig — Relative demand for chest imaging of outpatients without suspected or confirmed coronavirus disease 2019 (COVID-19) per day and the relative number of confirmed regional COVID-19 cases per day displayed as the percentage of the total number of requests for imaging (n = 1439) and reported cases (n = 5053) between February 27th 2020 and May 27th 2020. (TIF) [file pone.0247686.s003.tif]

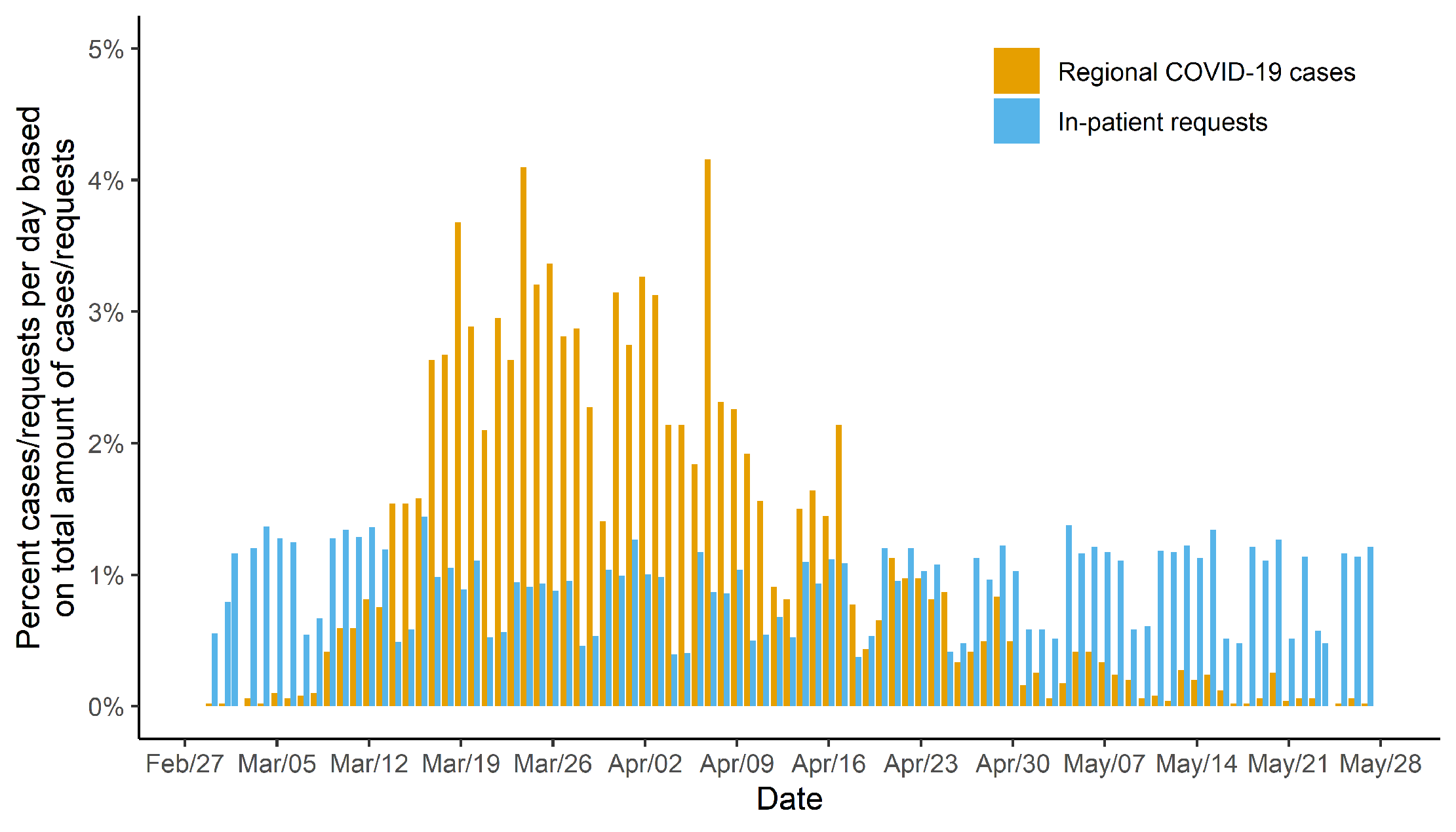

Supplement: S3 Fig — Relative demand for chest imaging of in-patients without suspected or confirmed coronavirus disease 2019 (COVID-19) per day and the relative number of confirmed regional COVID-19 cases per day displayed as the percentage of the total number of requests for imaging (n = 8123) and reported cases (n = 5053) between February 27th 2020 and May 27th 2020. (TIF) [file pone.0247686.s004.tif]
